# Nirmatrelvir and ritonavir for inpatients with severe or critical COVID-19 beyond five days of symptom onset: a propensity score-matched, multicenter, retrospective cohort study

**DOI:** 10.1186/s12879-024-09150-1

**Published:** 2024-06-18

**Authors:** Huan Zhang, Xiaojiao Tan, Zheng Zhang, Chenxi Wang, Haiqing Shi, Yao Li, Jianbo Li, Yan Kang, Xiaodong Jin, Xuelian Liao

**Affiliations:** 1 Department of Critical Care Medicine, West China Hospital, Sichuan University, 37 Guo Xue Xiang Street, Chengdu, Sichuan 610041 China; 2 Department of Cardiac Vascular Surgery Critical Care Medicine, The Third People’s Hospital of Chengdu, Chengdu, China; 3 Department of Critical Care Medicine, West China Tianfu Hospital of Sichuan University, Chengdu, China

**Keywords:** COVID-19, Nirmatrelvir and ritonavir, Severe, Critical

## Abstract

**Background:**

There is an urgent need for therapeutic strategies for inpatients with severe or critical COVID-19. The evaluation of the clinical benefits of nirmatrelvir and ritonavir (Nmr/r) for these patients beyond five days of symptom onset is insufficient.

**Methods:**

A new propensity score-matched cohort was constructed by using multicenter data from 6695 adult inpatients with COVID-19 from December 2022 to February 2023 in China after the epidemic control measures were lifted across the country. The severity of disease of the inpatients was based on the tenth trial edition of the Guidelines on the Diagnosis and Treatment of COVID-19 in China. The symptom onset of 1870 enrolled severe or critical inpatients was beyond five days, and they received either Nmr/r plus standard treatment or only standard care. The ratio of patients whose SOFA score improved more than 2 points, crucial respiratory endpoints, changes in inflammatory markers, safety on the seventh day following the initiation of Nmr/r treatment, and length of hospital stay were evaluated.

**Results:**

In the Nmr/r group, on Day 7, the number of patients with an improvement in SOFA score ≥ 2 was much greater than that in the standard treatment group (*P* = 0.024) without a significant decrease in glomerular filtration rate (*P* = 0.815). Additionally, the rate of new intubation was lower (*P* = 0.004) and the no intubation days were higher (*P* = 0.003) in the first 7 days in the Nmr/r group. Other clinical benefits were limited.

**Conclusions:**

Our study may provide new insight that inpatients with severe or critical COVID-19 beyond five days of symptom onset benefit from Nmr/r. Future studies, particularly randomized controlled trials, are necessary to verify the above findings.

**Supplementary Information:**

The online version contains supplementary material available at 10.1186/s12879-024-09150-1.

## Background

Severe acute respiratory syndrome coronavirus 2 (SARS-CoV-2), the cause of the COVID-19 pandemic, continues to threaten global health. A total of 690 million cases have been identified, and 36,088 individuals are still in severe or critical condition at present [1]. Nirmatrelvir and ritonavir (Nmr/r), as a SARS-CoV-2 protease inhibitor, has been authorized or supported for use worldwide for mild to moderate COVID-19. The EPIC-HR study demonstrated that adults at risk for severe COVID-19 benefited from the initiation of Nmr/r within five days of symptom onset [2]. Additionally, the Chinese National Medical Products Administration authorized the emergency prescription of Nmr/r on February 14, 2022. Patients infected with SARS-CoV-2 who need hospitalization often suffer severe comorbidities, multiple organ dysfunction, and even death [3]. In particular, the mortality of inpatients with severe or critical COVID-19 is high, which makes therapeutic strategies an urgent concern [4]. 

In China, SARS-CoV-2 epidemic control measures were lifted at the end of last year. The disease course of inpatients often exceeded five days after symptom onset. Additionally, new treatment options are crucial and necessary for inpatients with COVID-19 who are in severe or critical condition. There is a paucity of knowledge about the safety and efficacy of Nmr/r among inpatients with severe or critical SARS-CoV-2 beyond five days of symptom onset. We evaluated the clinical benefits of the late use of Nmr/r for inpatients with severe or critical SARS-CoV-2 in this study.

## Methods

### Study design and data source

All study procedures adhered to the Declaration of Helsinki. This study was approved by the Ethics Committee of West China Hospital (Approval No. (2023-20)). Upon admission, all the patients had signed informed consent forms for the use of their anonymized clinical data for future scientific research purposes. We retrieved all patient data from the medical records database, and the information department anonymized all the data involved before this study initiation.

We obtained our data from West China Hospital and West China Tianfu Hospital, two major referral centres for patients in Western China. A total of 6695 SARS-CoV-2-infected inpatients, confirmed by reverse-transcription polymerase chain reaction assay, were screened. The study period was from 7 Dec 2022 to 1 Feb 2023.

The inclusion criteria were as follows: (1) ≥ 18 years of age and ≥ 40 kg in weight; (2) confirmed SARS-CoV-2 test results (including home antigen test results); (3) disease course beyond five days at hospital admission; and (4) symptoms consistent with severe-to-critical COVID-19. The course of the disease was defined as the time from symptom onset to the first day of hospitalization. The definition of symptoms consistent with severe or critical COVID-19 was based on the tenth trial edition of the Guidelines on the Diagnosis and Treatment of COVID-19 in China: [5] (1) panting with a respiratory rate ≥ 30 beats/min; (2) SpO2 ≤ 93% without oxygen support at rest; (3) an oxygenation index ≤ 300 mmHg; (4) progressively worsening clinical symptoms and significant progression of internal lesions on lung imaging > 50% within 24 to 48 h; (5) breathing failure; (6) mechanical ventilation support; (7) shock; and (8) other organ failure requiring intensive care. We excluded patients according to the following criteria: (1) incomplete information on disease course, treatment, or diagnosis; (2) initiated Nmr/r more than 15 days after symptom onset, as some of the patients presented prolonged viral shedding and viral rebound; (3) had known or suspected renal or hepatic failure; (4) had a dialysis or glomerular filtration rate (GFR) < 30 ml/min/1.73 m^2^ within the past six months; (5) had contraindications for medications or allergies to Nmr/r ingredients; and (6) had used other antiviral treatments.

The patient information included comorbidities, Sequential Organ Failure Assessment (SOFA) score [6], and other clinical and laboratory examinations taken from the day of initial Nmr/r therapy to the next seven days. Day 1 was defined as the first day of hospitalization. All the above mentioned variables were the worst data points during a 24 h period on each day.

### Cohort definition

After admission, all patients with COVID-19 received standard treatment based on the Guidelines on the Diagnosis and Treatment of COVID-19 (Tenth Trial Edition). In the Nmr/r group, patients received standard treatment for COVID-19 and also were prescribed 300 mg of nirmatrelvir + 100 mg of ritonavir orally or 150 mg of nirmatrelvir + 50 mg of ritonavir every 12 h for 5 days if the GFR was within the 45 to 30 ml/min/1.73 m^2^ range. In the standard treatment group, patients received only standard treatment for COVID-19 according to the established guidelines. After preliminary analysis, a propensity score-matched cohort was constructed.

### Outcomes

The SOFA score was related to the evolution of clinical conditions. The primary outcome of our study was the percentage of patients whose SOFA score improved more than 2 (≥ 2) points on the seventh day following the initiation of Nmr/r treatment. Patients whose SOFA score decreased by less than 2 (< 2) points on Day 7 were categorized as not showing improvement. The variables of SOFA score can reflect the clinical severity of COVID-19 patients [7] and includes respiratory (PaO2/FiO2, mmHg), coagulation (platelets*10^9/L), cardiovascular (hypotension), liver (bilirubin, µmol/L), central nervous system (Glasgow Coma Scale), and renal (creatinine, µmol/L or urine output, ml/d) items. (Supplementary Table 1). An improvement in the SOFA score included any changes in the above variables.

Secondary outcomes were crucial respiratory endpoints seven days after the initiation of Nmr/r treatment including the rate of new intubation, new noninvasive ventilator (NIV) support, and new high-flow oxygen therapy, and no intubation days. We also analyzed the changes in inflammatory markers and drug-related side effects on Day 7, and the whole hospital stay.

### Statistical analysis

In the study cohort, the means and standard deviations were used to describe normally distributed quantitative data; otherwise, the medians and interquartile ranges were used. The numbers of cases and proportions were used to describe qualitative data.

The prescription of Nmr/r may have been biased by the patient’s clinical condition and associated factors, which could have led to covariant vectors between the two groups. To eliminate those covariates, we used a new propensity score-matched cohort to eliminate the main differences in baseline traits between the two groups.

The propensity score was obtained using the age-adjusted Charlson Comorbidity Index (age-CCI score), body mass index (BMI), disease course, vaccination status, equivalent steroid dose, antibiotics, disease-modifying agents (tocilizumab, baricitinib, and immunoglobulin), coinfection, oxygenation index, type of oxygen support, percentage of chest X-ray involvement, highest SOFA score, C-reactive protein (CRP) level and blood lymphocyte count on Day 1. The oxygenation indices were divided into 5 levels. An oxygenation index ≥ 400 represented to level 0, oxygenation index ≥ 300 and < 400 represented to level 1, oxygenation index ≥ 200 and < 300 represented to level 2, oxygenation index ≥ 100 and < 200 represented to level 3, and oxygenation index < 100 represented to level 4. Additionally, we focused on the features of chest CT images closely related to COVID-19, which were defined in a previous study [8]. To obtain a consensus on chest images, a blinded clinician and expert radiologist reviewed the chest CT scans independently. If there was disagreement, a third expert radiologist assisted in the judgment. The percentage of chest CT involvement was divided into 5 quartiles from 0 to 4 by 25% [9]. All the factors above mainly influenced the therapeutic method and the clinical results. The value of the caliper was less than 0.02, which revealed a negligible imbalance. In the new propensity score-matched cohort, the baseline features in both groups were as similar as possible for final multiple regression, except for Nmr/r which targeted the COVID-19 virus. All the analyses were conducted in IBM SPSS Statistics (25th Version). A *P* < 0.05 (2-sided) indicated statistical significance.

## Results

### Patients

**Fig. 1:**
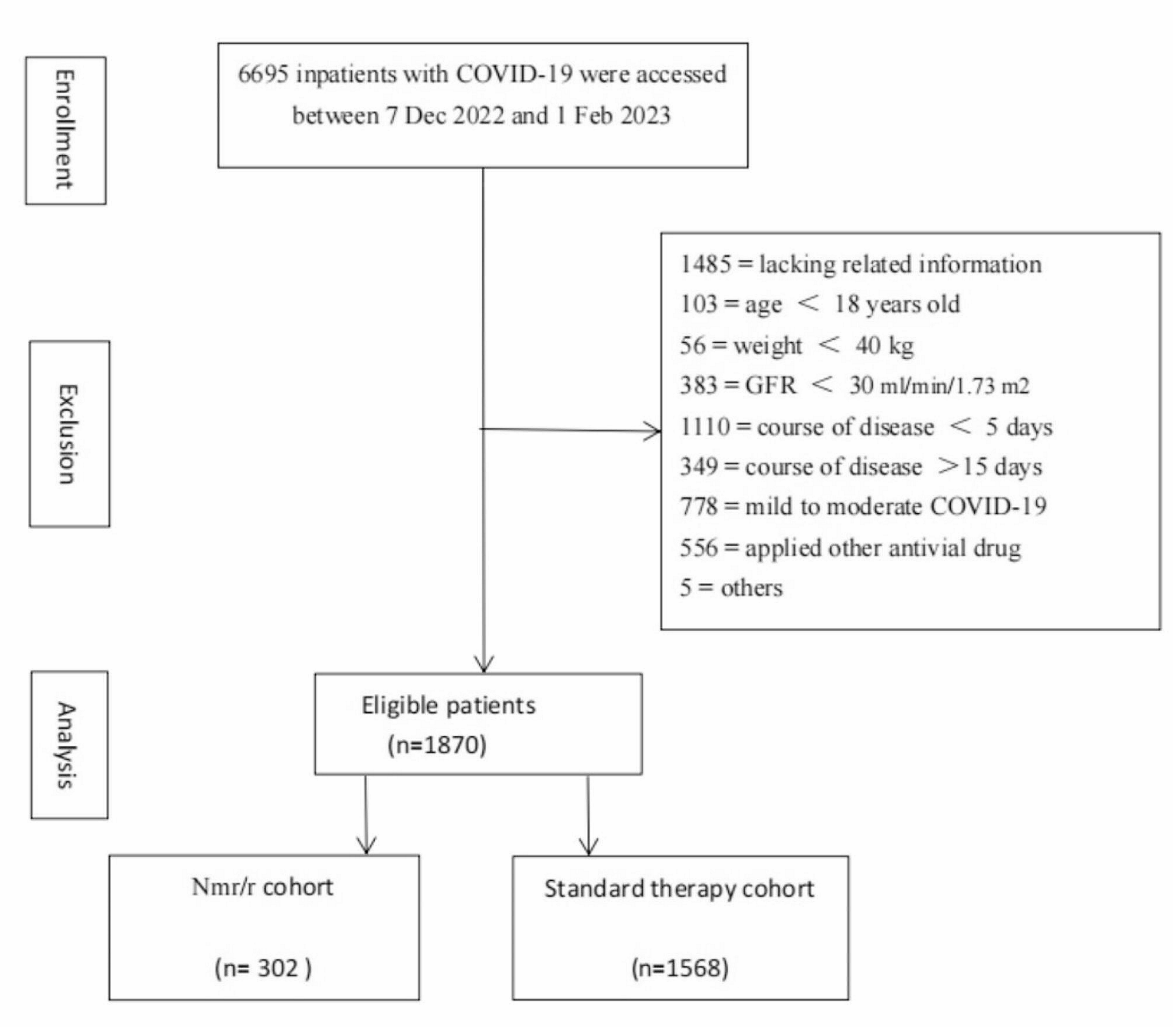
Flowchart of eligible inpatients

Of the 6695 patients screened, 1870 patients met the inclusion criteria at two large medical centres from 7 December 2022 to 1 February 2023. As some of the patients presented with prolonged viral shedding and viral rebound, we focused on the patients whose disease course ranged from 5 to 15 days in this study. Of the recruited severe or critical patients, a total of 302 patients received Nmr/r therapy plus standard treatment, and 1568 patients received standard treatment (Fig. 1).

### Baseline characteristics

The baseline information of all eligible patients in the two groups at admission before and after PSM is shown in Table 1. Before PSM, there were differences in baseline parameters between the two arms. Compared to those in the standard treatment cohort, the age-CCI score was much higher (4.12 ± 2.57 vs. 4.73 ± 2.41, *P* < 0.001) in Nmr/r therapy plus standard treatment patients. The prevalence of underlying health conditions showed significant differences between late Nmr/r recipients and nonrecipients. Additionally, significantly fewer patients in Nmr/r cohort had been vaccinated. What is more, the late Nmr/r therapy plus standard treatment patients had more severe conditions with higher SOFA scores (2.71 ± 2.70 vs. 3.67 ± 2.23, *P* < 0.001) and higher CPR levels [60.61 (22.01, 70.90) VS. 60.61 (24.23, 95.07), *P* = 0.004] than that in the control group at baseline. Moreover, other specific therapeutic strategies for COVID-19 were imbalanced between the two groups. In the new propensity score-matched cohort, the important covariates were similar between the two arms. (Supplementary Table 2).

**Table 1:** Demographic and clinical characteristics of 1870 eligible patients

Characteristic	Original cohort			PSM cohort		
	Standard treatment group (*N* = 1568)	Nmr/r group (*N* = 302 )	P value	Standard treatment group (*N* = 287)	Nmr/r group (*N* = 302)	P value
**Age (Mean ± SD) - yr**	65.22 ± 17.38	69.49 ± 16.99	< 0.001	67.65 ± 17.47	69.49 ± 16.99	0.195
**BMI**	23.52 ± 3.15	23.82 ± 2.75	0.113	23.78 ± 2.97	23.82 ± 2.75	0.820
**Male**	966 (61.6%)	214 (70.9%)	0.002	196 (68.3%)	214 (70.9%)	0.498
**Duration since symptom onset** **Median (range) - days**	10 (7,13)	9 (7, 13)	0.089	10 (7,13)	9 (7,13)	0.550
**Age-CCI score**	4.12 ± 2.57	4.73 ± 2.41	< 0.001	4.66 ± 2.47	4.73 ± 2.40	0.488
**Vaccination (N, %)**						
Received 1 dose	1519 (96.9%)	283 (93.7%)	0.007	2 (0.7%)	1 (0.3%)	0.072
Received 2 dose	1515 (96.6%)	282 (93.4%)	0.008	5 (1.7%)	3(1.0%)	0.130
Received 3 dose	1503 (95.9%)	279 (92.4%)	0.009	271 (94.3%)	279 (92.4%)	0.319
**Comorbidity (N, %)**						
Heart dysfunction	352 (22.4%)	84 (27.8%)	0.005	79 (27.5%)	84 (27.8%)	0.938
Respiratory dysfunction	403 (25.7%)	84 (27.8%)	0.444	70 (24.4%)	84 (27.8%)	0.345
Liver dysfunction	249 (15.9%)	55 (18.2%)	0.315	48 (16.7%)	55 (18.2%)	0.635
Kidney dysfunction	201 (12.8%)	48 (15.9%)	0.150	57 (19.9%)	48 (15.9%)	0.209
Malignancy	291 (18.6%)	36 (11.9%)	0.005	44 (15.3%)	36 (11.9%)	0.227
Hypertension	559 (35.7%)	146 (48.3%)	< 0.001	129 (44.9%)	146 (48.3%)	0.409
Diabetes mellitus	360 (23.0%)	107 (35.4%)	< 0.001	87 (30.3%)	107 (35.4%)	0.187
**Coinfection (N, %)**						
Bacteria	404 (25.8%)	129 (42.7%)	< 0.001	131 (45.6%)	129 (42.7%)	0.474
Candida	281 (17.9%)	86 (28.5%)	< 0.001	84 (29.3%)	86 (28.5%)	0.832
**Treatment** (N, %)						
Antibiotic	1245 (79.4%)	287 (95.0%)	< 0.001	266 (92.7%)	287 (95.0%%)	0.234
Steroids	726 (46.3%)	255 (84.4%)	< 0.001	230 (80.1%)	255 (84.4%)	0.172
Tocilizumab	33 (2.1%)	20 (6.6%)	< 0.001	18 (6.3%)	20 (6.6%)	0.863
Baricitinib	24 (1.5%)	12 (4.0%)	0.005	13 (4.5%)	12 (4.0%)	0.738
Immunoglobulins	93 (5.9%)	68 (22.5%)	< 0.001	67 (23.3%)	68 (22.5%)	0.811
**the type of oxygen support** (N, %)						
High flow oxygen	28 (1.8%)	10 (3.3%)	0.085	10 (3.5%)	10 (3.3%)	0.908
Noninvasive mechanical ventilation	74 (4.7%)	38 (12.6%)	< 0.001	18 (6.3%)	38 (12.6%)	0.009
Invasive mechanical ventilation	62 (4.0%)	15 (5.0%)	0.417	18 (6.3%)	15 (5.0%)	0.491
**SOFA score on Day 1** **(Mean ± SD)**	2.71 ± 2.70	3.67 ± 2.23	< 0.001	3.41 ± 3.13	3.67 ± 2.23	0.001
**CRP level on Day 1** **(Median (range) - days**	60.61 (22.01, 70.90)	60.61 (24.23, 95.07)	0.004	60.61 (22.50, 81.20)	60.61 (24.30, 95.00)	0.218
**Level of PO2/FIO2**			< 0.001			< 0.001
Level 0	543 (34.6%)	51 (16.9%)		77 (26.8%)	51 (16.9%)	
Level 1	417 (26.6%)	35 (11.6%)		53 (18.5%)	35 (11.6%)	
Level 2	188 (12.0%)	39 (12.9%)		32 (11.1%)	39 (12.9%)	
Level 3	277 (17.7%)	122 (40.4%)		73 (25.4%)	122 (40.4%)	
Level 4	143 (9.1%)	55 (18.2%)		52 (18.1%)	55 (18.2%)	
**the percentage of chest CT involvement**			< 0.001			0.286
0%	12 (1.2%)	0 (0.0%)		1 (0.3%)	0(0.0%)	
< 25%	507 (49.2%)	143 (47.4%)		108 (37.6%)	143 (47.4%)	
≥ 25%, < 50%	29 (2.8%)	11 (4.2%)		71 (24.7%)	50 (16.6%)	
≥ 50%, < 75%	163 (15.8%)	56 (21.3%)		57 (19.9%)	56 (18.5%)	
≥ 75%	170 (16.5%)	53 (20.2%)		50 (17.4%)	53 (17.5%)	

Abbreviations: SD: standard deviation; age-CCI: age-adjusted Charlson Comorbidity Index; BMI: Body Mass Index; SOFA: Sequential Organ Failure Assessment; CRP: C-reactive protein;

Categorical variables were compared by chi-squared tests; Continuous variables were compared by independent-samples T test or nonparametric test

### Primary outcome

After 7 days of therapy, 503 patients achieved an improvement in SOFA score ≥ 2. The ratio of patients with an improvement in SOFA score that decreased by more than 2 points was higher in the Nmr/r plus standard therapy group than that in the standard therapy group (37.1% vs. 24.9%). In the new propensity score-matched cohort, the heterogeneous covariates were eliminated. The degree of decrease in SOFA scores in the Nmr/r group and in the standard therapy group were − 0.60 ± 3.09 and 0.09 ± 3.52, respectively. The degree of decrease in SOFA score was significantly higher in the Nmr/r group than that in the standard therapy group (*P* = 0.012). According to the adjusted regression analysis, the ratio of Nmr/r plus standard care recipients with an improvement in SOFA score ≥ 2 was also notably higher (*P* = 0.024), and the OR (95% CI) was 1.576 (1.062–2.337), as shown in Table 2.

**Table 2:** After 7 days of therapy, the rate of inpatients with SOFA score improvement ≥ 2

Item	PSM Cohort				Original cohort			
	Patients with improvement^a^	Patients without improvement^b^	P value	Adjusted OR (95% CI)	Patients with improvement ^a^	Patients without improvement ^b^	P value	Adjusted OR (95% CI)
Nmr/r plus standard therapy	112 (60.2%)	190 (47.1%)	0.024*	1.576 (1.062–2.337)	112 (37.1%)	391 (24.9%)	0.114	1.292 (0.940–1.774)
Age-CCI	4.76 ± 2.35	4.66 ± 2.47	0.217	0.948 (0.870–1.032)	4.39 ± 2.41	4.15 ± 2.60	0.050	0.952 (0.906-1.000)
BMI	23.94 ± 2.56	23.74 ± 2.98	0.777	0.990 (0.922–1.063)	23.72 ± 2.97	23.51 ± 3.13	0.888	0.997 (0.960–1.036)
Course	9.00 (7.00, 13.00)	9.00 (7.00, 13.00)	0. 579	0.983 (0.924–1.045)	10 (7.00, 13.00)	10 (7.00, 13.00)	0.901	1.002 (0.966–1.040)
Vaccination			0.870	1.025 (0.766–1.370)			0.452	0.930 (0.771–1.123)
Received 1 dose	1 (0.5%)	2 (0.5%)			1 (0.2%)	4 (0.9%)		
Received 2 dose	3 (1.6%)	5 (1.2%)			3 (0.6%)	12 (0.9%)		
Received 3 dose	173 (93.0%)	377 (93.5%)			474 (94.2%)	1308 (95.7%)		
Level of PO2/FIO2 on Day 1			< 0.001*	1.699 (1.440–2.005)			< 0.001*	1.8883 (1.698–2.088)
Level 0	13 (7.0%)	115 (28.5%)			47 (9.3%)	547 (40.0%)		
Level 1	12 (6.5%)	76 (18.9%)			73 (14.5%)	379 (27.7%)		
Level 2	33 (17.7%)	38 (9.4%)			109 (21.7%)	118 (8.6%)		
Level 3	89 (47.8%)	106 (26.3%)			196 (39.0%)	203 (14.9%)		
Level 4	39 (21.0%)	68 (16.9%)			78 (15.5%)	120 (8.8%)		
Oxygen support on Day 1			< 0.001*	0.395 (0.290–0.538)			< 0.001*	0.358 (0.289–0.444)
High flow oxygen	5 (2.7%)	15 (3.7%)			8 (1.6%)	30 (2.2%)		
NIV	10 (5.4%)	46 (11.4%)			18 (3.6%)	94 (6.9%)		
IMV	3 (1.6%)	30 (7.4%)			9 (1.8%)	68 (5.0%)		
Level of chest CT on admission			0.441	0.935 (0.788–1.109)			0.059	0.896 (0.799–1.004)
0%	0 (0.0%)	1 (0.2%)			1 (0.2%)	11 (0.8%)		
< 25%	92 (49.5%)	159 (39.5%)			259 (51.5%)	540 (39.5%)		
≥ 25%, < 50%	25 (13.4%)	96 (23.8%)			100 (19.9%)	517 (37.8%)		
≥ 50%, < 75%	39 (21.0%)	74 (18.4%)			75 (14.9%)	144 (10.5%)		
≥ 75%	30 (16.1%)	73 (18.1%)			68 (13.5%)	155 (11.3%)		
Equivalent steroid dose	200.00 (125.00, 200.00)	125.00 (125.00, 200.00)	0.397	0.999 (0.996–1.002)	125.00 (0.00, 125.00)	125.00 (0.00, 200.00)	0.026*	0.998 (0.997-1.000)
Antibiotic (N, %)	172 (92.5%)	381 (94.5%)	0.117	0.519 (0.228–1.180)	443 (88.1%)	1089 (79.7%)	0.801	1.046 (0.735–1.489)
Tocilizumab	13(7.0%)	25(6.2%)	0.887	1.059 (0.478–2.344)	13 (2.6%)	23 (1.7%)	0.687	1.154 (0.587–2.266)
Baricitinib	10(5.4%)	15(3.7%)	0.845	1.097(0.435–2.764)	21 (4.2%)	32 (2.3%0	0.618	0.819 (0.373–1.797)
Immuoglobulins	42(22.6)	93(23.1%)	0.478	1.193(0.732–1.944)	48(9.5%)	113 (8.3%)	0.720	0.924 (0.600-1.423)
Bacterium (N, %)	76 (40.9%)	184 (45.7%)	0.222	0.710 (0.409–1.230)	155 (30.8%)	378 (27.7%)	0.240	0.819 (0.588–1.142)
Candida (N, %)	54 (29.0%)	116 (28.8%)	0.633	1.155 (0.640–2.086)	116 (23.1%)	251 (18.4%)	0.615	1.099 (0.760–1.589)
SOFA score on Day 1	4.25 ± 2.02	3.22 ± 2.92	< 0.001*	1.156 (1.066–1.253)	4.08 ± 2.18	2.41 ± 2.68	< 0.001*	1.247 (1.184–1.314)
CRP level on Day 1	60.61 (21.35, 84.00)	60.61 (21.35, 98.50)	0.056	1.004 (1.000-1.008)	60.61 (22.50, 68.80)	60.61 (22.03, 90.8)	0.239	1.002 (0.999–1.004)
Lymphocyte level on Day 1	1.27 ± 0.769	1.22 ± 0.789	0.195	1.185 (0.917–1.530)	1.31 ± 0.72	1.38 ± 0.20	0.537	1.049 (0.901–1.222)

Abbreviations: age-CCI: age-adjusted Charlson Comorbidity Index; BMI: Body Mass Index; SOFA: Sequential Organ Failure Assessment; CRP: C-reactive protein; NIV: Noninvasive mechanical ventilation; IMV: Invasive mechanical ventilation

^a^Patients whose SOFA score decreased by more than 2 (≥ 2 ) points on Day 7

^b^Patients whose SOFA score decreased by less than 2 (< 2) points on Day 7

*With significance and *P* ≤ 0.05

In the new propensity score matched cohort, the heterogeneous covariates were eliminated. Using the adjusted regression to analysis the primary outcomes

Other items at baseline also independently influenced odds of clinical improvement in SOFA score ≥ 2 on Day 7, including (1) PO2/FIO2, which had an OR (95% CI) of 1.699 (1.440–2.005); (2) Oxygen support, which had an OR (95% CI) of 0.395 (0.290–0.538); and (3) SOFA score, which had an OR (95% CI) of 1.156 (1.066–1.253).

### Secondary outcomes

It is crucial to concentrate on respiratory endpoints in the first 7 days after the initiation of therapy for COVID-19. The incidence of new-onset acute respiratory distress syndrome (ARDS) was 4.4%, and the rate of new high-flow oxygen support, NIV, and intubation were 53%, 5.3%, and 4.7%, respectively. In the new propensity score-matched cohort, among severe or critical adult patients with SARS-CoV-2 infection, initiating Nmr/r plus standard therapy beyond 5 days of symptom onset significantly decreased the rate of new in the first 7 days, with an OR (95% CI) of 0.222 (0.080–0.610). Additionally, Nmr/r significantly increased the no intubation days in the first 7 days after the initiation of therapy for COVID-19 (*P* = 0.003), and the difference in days and 95% CI was 0.237 (0.082–0.392), as shown in Table 3.

**Table 3:** The effect of late using Nmr/r on the new type of oxygen support, the rate of new-onset ARDS and no intubation days in the first 7 days after initiation of therapy for COVID-19

Item	PSM Cohort				Original cohort			
	Standard group	Nmr/r group	P value	Adjusted OR (95% CI)	Standard group	Nmr/r group	P value	Adjusted OR (95% CI)
The rate of new intubation N (%)	27 (9.4%)	17 (5.6%)	0.004*	0.222 (0.080–0.610)	70 (4.5%)	17 (5.6%)	0.118	0.469 (0.182–1.210)
The rate of new NIV support N (%)	23 (8.0%)	29 (9.6%)	0.953	0.980 (0.504–1.907)	71 (4.5%)	29 (9.6%)	0.897	1.040 (0.577–1.873)
The rate of new high flow oxygen support N (%)	26 (9.1%)	31 (10.3%)	0.735	1.107 (0.616–1.989)	68 (4.3%)	31 (10.3%)	0.161	1.417 (0.870–2.307)
The rate of new-onset ARDS N (%)	14 (4.9%)	14 (4.6%)	0.704	1.184 (0.495–2.831)	69 (4.4%)	14 (4.6%)	0.613	1.201 (0.590–2.443)
No intubation days (M ± SD)	6.62 ± 1.33	6.77 ± 1.11	0.003*	0.237 (0.082–0.392	6.81 ± 0.97	6.77 ± 1.11	0.018*	0.124 (0.021–0.227)

Abbreviations: M ± SD: Mean ± standard deviation; PSM cohort: propensity score matched cohort; NIV: noninvasive mechanical ventilation; ARDS: Acute Respiratory Distress Syndrome

*With significance and *P* ≤ 0.05

In the new propensity score matched cohort, the heterogeneous covariates were eliminated. Using the adjusted regression to analysis the secondary outcomes

**Fig. 2:**
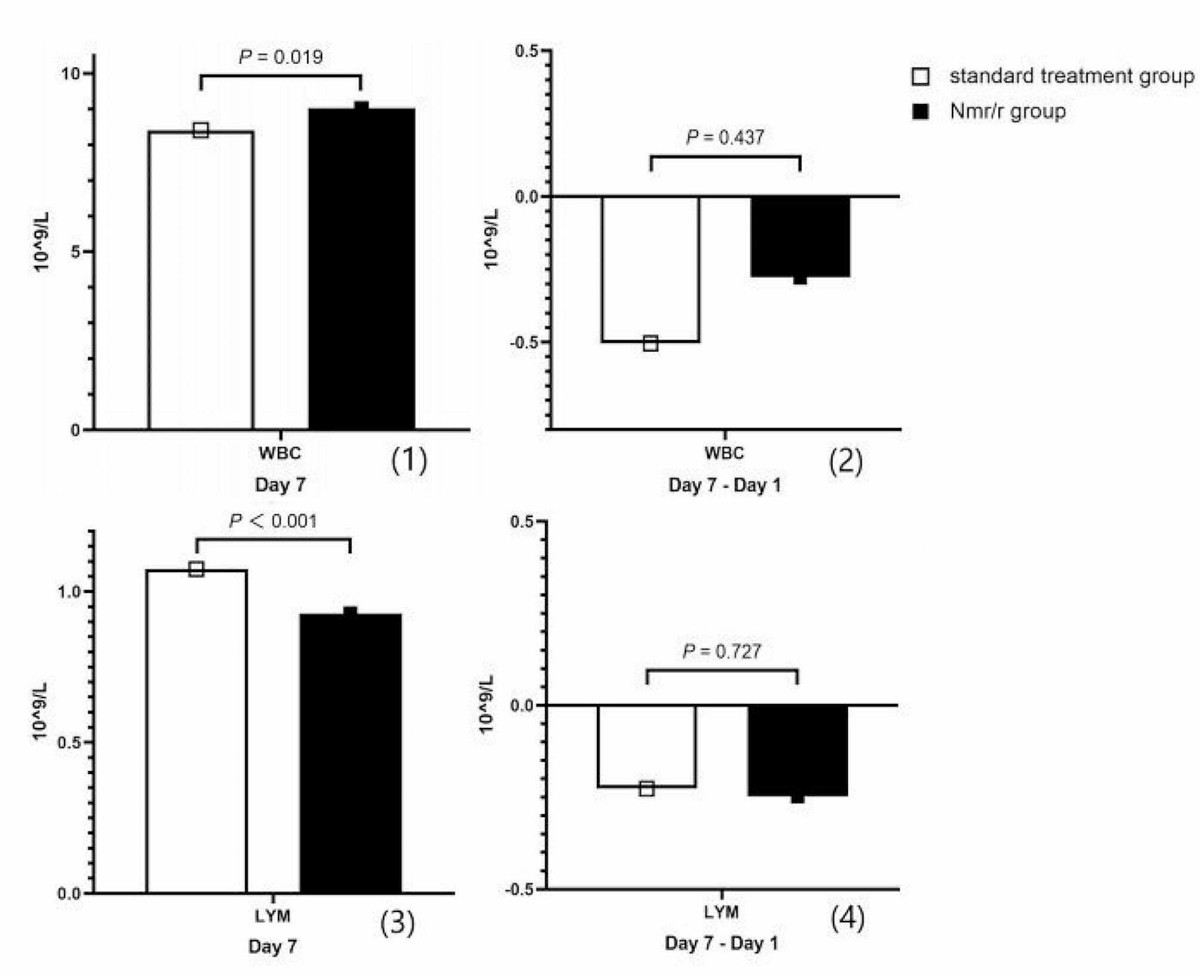
The level of WBC and blood LYM counts on Day 7 and the degree of changes of WBC and blood LYM counts (Day 7- Day 1) between the two group. Abbreviations: WBC: white blood cell; LYM: lymphocyte. In the new propensity score matched cohort, the heterogeneous covariates were eliminated. After 7 days, the WBC level was significantly higher in Nmr/r group (9.03 ± 3.48 VS. 8.42 ± 2.89) and the blood LYM level was significantly lower in Nmr/r group (0.93 ± 0.52 VS. 1.07 ± 0.44). However, the degree of changes of the WBC and blood lymphocyte level were not notably different between the Nmr/r group and the standard treatment group, and the mean ± SD were − 0.28 ± 3.69 VS. -0.50 ± 3.69 and − 0.25 ± 0.68 VS. -0.23 ± 0.75, respectively

The CRP level on the Day 7 was not significantly lower in the Nmr/r group [16.44 (6.91, 19.75) vs. (19.75 (9.96, 19.75), *P* = 0.073) and the degree of decreased CRP level was not notably greater in the Nmr/r plus standard treatment group than that in the standard treatment group [-40.86 (-78.91, -12.33) vs. -40.86 (-63.50, -8.40), *P* = 0.054]. We also focused on the changes in the levels of other inflammatory markers, including white blood cell (WBC) and blood lymphocyte (LYM) counts, on Day 7. Compared to those in the standard treatment group, the WBC count was greater (*P* = 0.019), and the blood LYM count was lower (*P* < 0.001) in the Nmr/r group. However, the changes in the WBC (*P* = 0.437) and blood LYM (*P* = 0.727) counts were not significantly different between the two groups (Fig. 2).

When exploring the effect of Nmr/r use on the length hospital stay, in the new propensity score-matched group, the length of hospital stay was longer in the late Nmr/r group, and the difference in days and the 95% CI were 1.880 (0.838–2.923). Further more, we excluded patients who died in the hospital because they could have influenced the overall length of hospital stay. However, the length of hospital stay was not significantly longer in the late Nmr/r group, and the difference in days and the 95% CI were 0.671 (-0.455-1.796)).

### Subgroup analysis

Subgroup analysis was performed according to age. A total of 651 patients were in the younger subgroup (< 60 years old). The older subgroup included 1219 patients who were older than 60 years. A total of 243 patients in the older subgroup received Nmr/r therapy. After PSM, compared to that in the whole cohort, the initiation of Nmr/r had a similar effect on the ratio of an improvement in SOFA score ≥ 2 in the older subgroup on Day 7 (OR (95% CI) = 1.559 (0.990–2.455)). Additionally, late use of Nmr/r significantly decreased the rate of new intubation in the first 7 days (OR (95% CI) = 0.152 (0.040–0.580)) in the older subgroup (Supplementary Tables 3 and 4). As the number of patients in the younger subgroup was relative limit, the efficacy of late use of Nmr/r in this subgroup was not verified which may be at risk of low-level evidence.

**Safety outcome**.

**Fig. 3:**
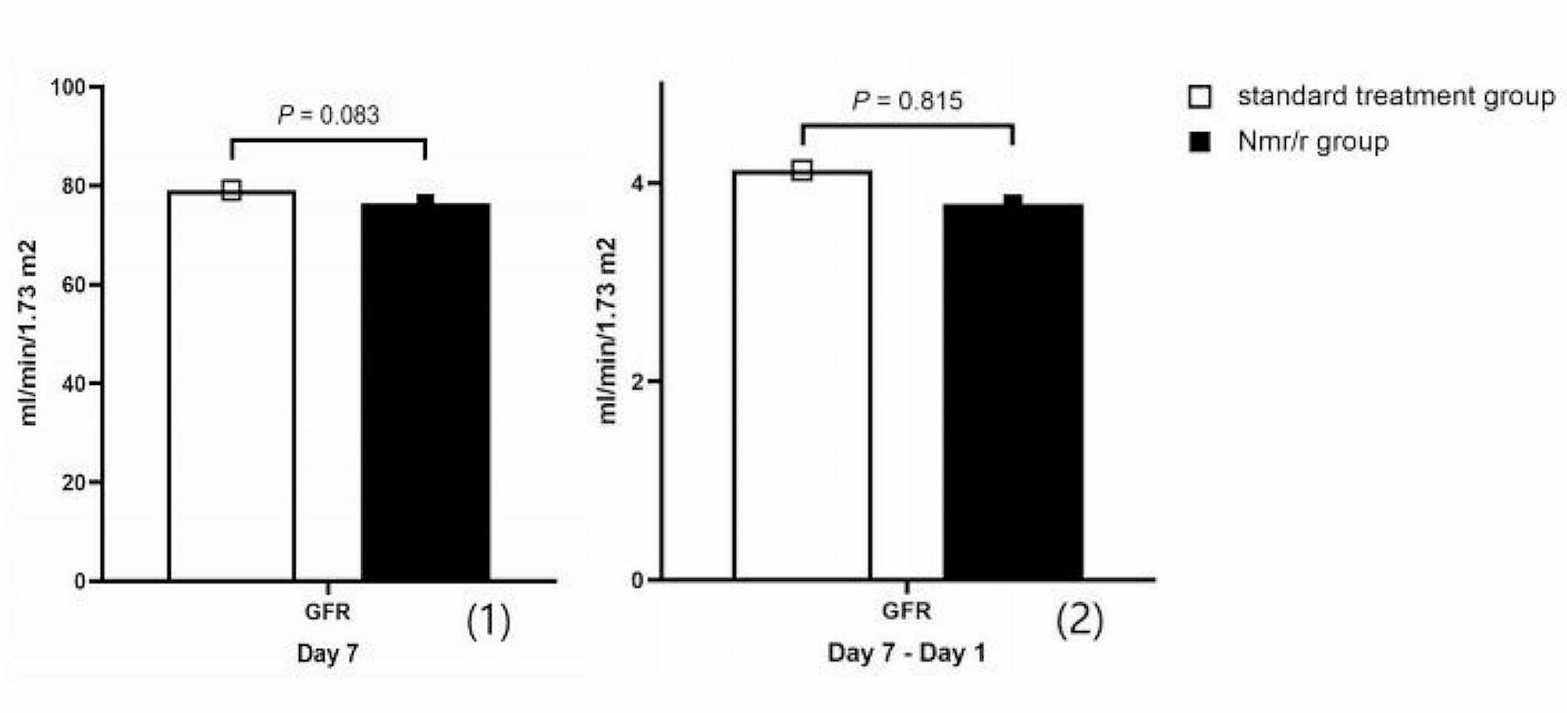
The level of blood GFR on Day 7 and the degree of change of blood GFR (Day 7- Day 1) between the two group. Abbreviations: GFR: glomerular filtration rate; Legend: In the new propensity score matched cohort, the heterogeneous covariates were eliminated. The blood GFR level was not significantly lower in Nmr/r group (76.40 ± 19.65 VS. 79.04 ± 17.16) on day 7. Also, the degree of change of the blood GFR level was not notably different between the Nmr/r group and the standard treatment group, and the mean ± SD was 3.79 ± 16.01 VS. 4.13 ± 19.07

After 7 days of therapy, the blood GFR was not significantly lower in the patients who received Nmr/r than in the patients who were treated only with standard therapy. Additionally, the degree of change was not significantly different between the two groups. These results are shown in Fig. 3.

## Discussion

By using a large amount of patient data from two major medical institutions in Western China, this real-world analysis demonstrated that initiating Nmr/r beyond 5 days of symptom onset improved the multiple organ dysfunction in severe or critical patients with COVID-19, as reflected by the SOFA score on Day 7. The results showed that the percentage of patients with an improvement in the SOFA score, indicated by a decrease of more than 2 points on Day 7 after the initiation of Nmr/r, was much greater than that in the standard treatment group. Importantly, the other variables at baseline that reflected disease severity and were independently associated with increased odds of an improvement in the SOFA score by more than 2 points included PO2/FIO2, oxygen support and SOFA score. This further indicated that initiating Nmr/r beyond 5 days of symptom onset was effective for severe or critical patients. Additionally, the late use of Nmr/r reduced the rate of new intubation and increased the no invasive mechanical ventilation days in the first 7 days. Among the surviving patients, the length of hospital stay was similar in both groups.

The use of Nmr/r was authorized by the Chinese National Medical Products Administration for adults suffering mild to moderate COVID-19 symptoms and patients at high risk of progression to severe conditions. Our study included inpatients with severe-to-critical conditions for several reasons. First, there are currently 22,092,160 actively infected COVID-19 patients. Additionally, 2% of the patients are in severe-to-critical condition worldwide. Second, the average incubation period of COVID-19 is 5.2 days [10]. In China, during the two months after SARS-CoV-2 control measures were lifted, most of the inpatients were in severe or critical condition, and the disease course was often more than five days. In this study, the average disease course was 10 days. The therapeutic options for all these patients involved challenges.

To date, studies of Nmr/r therapy among inpatients with severe to critical COVID-19 have been limited. The baseline SOFA score in our study was 2.68 ± 0.61. As Christopher W. Seymour reported, the risk of death among patients with suspected infection with SOFA scores ≥ 2 was 2–25 times that of patients with SOFA scores less than 2 [11]. A high SOFA score is also a potential risk factor for a poor COVID-19 prognosis [12]. Our real-world analysis demonstrated that after 7 days of therapy, the degree of decrease in SOFA score was significantly greater in the Nmr/r group than that in the standard therapy group.

In our study, the late use of Nmr/r decreased the new intubation rate in the first 7 days, especially among elderly patients. The study by Weng [13] also demonstrated that the use of Nmr/r was beneficial for elderly COVID-19 patients without adverse events. Our results were also similar to those of the EPIC-HR study [2]. Additionally, a case report indicated that extending the therapeutic course of Nmr/r might be a feasible and safe strategy for patients with severe COVID-19 [14]. Jiao Liu [15] showed that the use of Nmr/r did not significantly reduce the risk of all-cause mortality on Day 28 among adult COVID-19 inpatients with severe comorbidities. Nmr/r may have different therapeutic effects at different stages of the disease among different inpatients. However, some differences from other studies may explain these discrepancies. First, the inpatients in our study were in severe to critical condition and needed much more urgent therapy to manage their disease. Although the patients in the study by Jiao Liu in China had severe comorbidities, none of the participants were in severe to critical condition. Moreover, the initiation time of Nmr/r in our research was more than 5 days after disease onset, which was different from that in the other two studies. A study by Wang [16] indicated that treatment with Nmr/r beyond five days after symptom onset had no obvious effect on viral elimination time compared with early treatment. However, no clinical outcome was discussed in his study.

Nmr/r is a potent inhibitor of cytochrome P450-3A4 (CYP3A4), and coadministration of other drugs may degrade this enzyme [17]. The standard therapy for severe to critical patients or patients with specific comorbidities, including anticoagulants, corticosteroids, and others, may impede the clinical efficacy of Nmr/r. Considering the above factors, larger prospective studies, particularly randomized controlled trials, need to be conducted to confirm these findings in different populations.

This study has several strengths. First, our study provides an important therapeutic reference for inpatients with severe or critical COVID-19 beyond 5 days after symptom onset. Second, therapeutic strategies for severe or critical inpatients are urgently needed. Finally, we created a new propensity score-matched cohort with a relatively large amount of data. The results of this study were relatively persuasive.

However, this study also has several limitations. First, this was a retrospective cohort study. Additionally, we did not include other factors that may have influenced the late initiation of Nmr/r or clinical outcomes. Second, in the new PSM cohort, the SOFA score and the PIO2/FIO2 were more severe in the Nmr/r group. However, the number of patients with an improved SOFA score by more than two points were much greater in the Nmr/r group and the rate of new tracheal intubation was much lower in the Nmr/r group. That is, late initiation of Nmr/r was effective for the patients in severe or critical condition. Moreover, as this was a retrospective study, we did not explore the association between the 14-day or 28-day incubation rate and the late use of Nmr/r, which may have led to serious bias. Additionally, as the relative small scale data in younger subgroup, the efficacy of late use of Nmr/r in this subgroup was not verified which may be at risk of low-level evidence. What is more, we used the improvement in the SOFA score as the primary outcome, which may be good for specific patients, but the long-term effect of Nmr/r reflected by the SOFA score was difficult to obtain due to highly heterogeneous assessments by different clinicians. Therefore, the results of our main outcomes may be at risk of low-level evidence.

## Conclusions

Our study may provide new insight that inpatients with severe or critical COVID-19 beyond five days of symptom onset benefit from Nmr/r. Future studies, such as larger prospective studies, particularly randomized controlled trials, are needed to verify the above findings.

### Electronic supplementary material

Below is the link to the electronic supplementary material.

Supplementary Material 1

## Data Availability

The datasets generated during and/or analysed during the current study are not publicly available due to all data from the electronic medical record system in the hospital which are not public, but are available from the corresponding author on reasonable request.

## Abbreviations

SARS-CoV-2: severe acute respiratory syndrome coronavirus 2
Nmr/r: Nirmatrelvir/ritonavir
GFR: glomerular filtration rate
SOFA score: Sequential Organ Failure Assessment score
SD: standard deviation
NIV: noninvasive mechanical ventilation
IMV: Invasive mechanical ventilation
ARDS: Acute Respiratory Distress Syndrome
age-CCI: age-adjusted Charlson Comorbidity Index
BMI: body mass index
CRP: C-reactive protein
WBC: white blood cell
LYM: lymphocyte
