# Supplementary material for: Nirmatrelvir and ritonavir for inpatients with severe or critical COVID-19 beyond five days of symptom onset: a propensity score-matched, multicenter, retrospective cohort study
Source: BMC Infect Dis. 2024 Jun 18;24:597. doi: 10.1186/s12879-024-09150-1 (PMC11184924; doi:10.1186/s12879-024-09150-1)
Supplement: Supplementary file 1 — Supplementary Material 1 [file 12879_2024_9150_MOESM1_ESM.docx]

Supplementary table 1. The Sequential Organ Failure Assessment score (SOFA score)

|  | SOFA score | | | | |
| --- | --- | --- | --- | --- | --- |
| Items | 0 | 1 | 2 | 3 | 4 |
| Respiratory  PaO2/FiO2, mmHg | ＞400 | ≤400 | ≤300 | ≤200 | ≤100 ^a^ |
| Coagulation  Platelets*10^9/L | ＞150 | ≤150 | ≤100 | ≤50 | ≤20 |
| Cardiovascular  Hypotension | No hypotension | MAP ≤70 mmHg | Dopamine ≤5 or  Dobutamine any dose | Dopamine ＞5 or  Epinephrine ≤0.1 or  Norepinephrine ≤0.1 | Dopamine ＞15 or  Epinephrine ＞0.1 or  Norepinephrine ＞0.1 |
| Liver  Bilirubin, μmol/L | ＜20 | 20-32 | 33-101 | 102-204 | ＞204 |
| Central nervous system  Glasgow Coma Scale | 15 | 13-14 | 10-12 | 6-9 | ＜6 |
| Renal  Creatinine, μmol/L  OR urine output | ＜110 | ＜110-170 | ＜171-299 | 300-440  ＜500ml/d | ＞440  ＜200ml/d |

a: Values are with respiratory support.

Supplementary Table 2. Baseline characteristics of covariates in the original cohort and in the new propensity score matched cohort, respectively.

| Covariate | Original cohort | | | | | PSM cohort | | | | |
| --- | --- | --- | --- | --- | --- | --- | --- | --- | --- | --- |
|  | Standard group N=1568 | Nmr/r group N=302 | *P* | Std. Mean Diff. | Var. Ratio | Standard group N=298 | Nmr/r group N=302 | *p* | Std. Mean Diff. | Var. Ratio |
| Age-CCI  mean±SD | 4.12±2.57 | 4.73±2.41 | ＜0.001 | 0.253 | 0.877 | 4.66±2.47 | 4.73±2.40 | 0.488 | -0.067 | 0.872 |
| BMI | 23.51±3.15 | 23.82±2.75 | 0.113 | 0.112 | 0.760 | 23.78±2.97 | 23.82±2.75 | 0.820 | -0.018 | 0.720 |
| Course | 10（7,13） | 9（7,13） | 0.089 | -0.111 | 1.023 | 10（7,13） | 9（7,13） | 0.550 | 0.037 | 1.163 |
| Dose of vaccination |  |  | 0.057 |  |  |  |  | 0.274 |  |  |
| 0 dose of vaccination | 49（3.1%） | 19（6.3%） |  | 0.130 |  | 10（3.4%） | 19（6.3%） |  | 0.031 |  |
| 1 dose of vaccination | 4（0.3%） | 1（0.3%） |  | 0.012 |  | 2（0.7%） | 1（0.3%） |  | 0.000 |  |
| 2 dose of vaccination | 12（0.8%） | 3（1.0%） |  | 0.023 |  | 5 (1.7%) | 3(1.0%) |  | -0.038 |  |
| 3 dose of vaccination | 1503（95.9%） | 279（92.4%） |  | -0.131 |  | 271(94.3%) | 279(92.4%) |  | -0.014 |  |
| Equivalent steroid dose （Median, IQR） | 125 (0,125) | 125 (125,200) | ＜0.001 | 0.925 | 0.661 | 200 (125,200) | 125(125,200) | 0.263 | -0.027 | 0.853 |
| Tocilizumab (N, %) | 33 (2.1%) | 20 (6.6%) | ＜0.001 | 0.182 |  | 18（6.3%） | 20（6.6%） | 0.863 | 0.075 |  |
| Baricitinib (N, %) | 24 (1.5%) | 12 (4.0%) | 0.005 | 0.125 |  | 13（4.5%） | 12（4.0%） | 0.738 | 0.000 |  |
| Immunoglobulins (N, %) | 93 (5.9%) | 68 (22.5%) | ＜0.001 | 0.397 |  | 67（23.3%） | 68 (22.5%) | 0.811 | 0.045 |  |
| Antibiotic (N, %) | 1245 (79.4%) | 287 (95.0%) | ＜0.001 | 0.720 |  | 266 (92.7%) | 287(95.0%%) | 0.234 | 0.000 |  |
| Bacterium (N, %) | 404 (25.8%) | 129 (42.7%) | ＜0.001 | 0.343 |  | 131(45.6%) | 129(42.7%) | 0.474 | 0.038 |  |
| Candida (N, %) | 281 (17.9%) | 86 (28.5%) | ＜0.001 | 0.234 |  | 84 (29.3%) | 86 (28.5%) | 0.832 | -0.008 |  |
| SOFA score  mean±SD | 2.71±2.70 | 3.67±2.23 | ＜0.001 | 0.434 | 0.680 | 3.41±3.13 | 3.67±2.23 | 0.001 | -0.091 | 0.434 |
| CRP level  （Median, IQR） | 60.61（22.01,70.90） | 60.61  （24.23，9.08） | 0.004 | 0.183 | 1.283 | 60.61  （22.50, 81.20） | 60.61  （24.30, 95.00） | 0.218 | 0.051 | 1.191 |
| Lymphocyte level mean±SD | 1.40±0.80 | 1.18±0.76 | ＜0.001 | -0.296 | 0.910 | 1.301±0.801 | 1.18±0.76 | 0.050 | -0.093 | 1.087 |
| Level of PO2/FIO2 |  |  | ＜0.001 |  |  |  |  | ＜0.001 |  |  |
| Level 0 | 543（34.6%） | 51（16.9%） |  | -0.474 |  | 77(26.8%) | 51(16.9%) |  | -0.010 |  |
| Level 1 | 417（26.6%） | 35（11.6%） |  | -0.474 |  | 53(18.5%) | 35(11.6%) |  | -0.023 |  |
| Level 2 | 188（12.0%） | 39（12.9%） |  | 0.028 |  | 32(11.1%) | 39(12.9%) |  | 0.045 |  |
| Level 3 | 277（17.7%） | 122（40.4%） |  | 0.463 |  | 73(25.4%) | 122(40.4%) |  | -0.015 |  |
| Level 4 | 143（9.1%） | 55（18.2%） |  | 0.237 |  | 52(18.1%) | 55(18.2%) |  | 0.001 |  |
| the type of oxygen support |  |  | ＜0.001 |  |  |  |  | 0.070 |  |  |
| High flow oxygen | 28（1.8%） | 10（3.3%） |  | 0.085 |  | 10(3.5%) | 10(3.3%) |  | 0.000 |  |
| NIV | 74（4.7%） | 38（12.6%） |  | 0.237 |  | 18(6.3%) | 38(12.6%) |  | -0.057 |  |
| IMV | 62（4.0%） | 15（5.0%） |  | 0.047 |  | 18(6.3%) | 15(5.0%) |  | 0.000 |  |
| the percentage of chest CT involvement |  |  | ＜0.001 |  |  |  |  | 0.286 |  |  |
| 0% | 12（1.2%） | 0 （0.0%） |  | -0.096 |  | 1 (0.3%) | 0(0.0%) |  | 0.000 |  |
| ＜ 25% | 507（49.2%） | 128（48.7%） |  | 0.110 |  | 108 (37.6%) | 143(47.4%) |  | 0.105 |  |
| ≥ 25%, ＜ 50% | 29（2.8%） | 11（4.2%） |  | -0.527 |  | 71 (24.7%) | 50(16.6%) |  | 0.061 |  |
| ≥ 50%, ＜ 75% | 163（15.8%） | 56（21.3%） |  | 0.210 |  | 57 (19.9%) | 56(18.5%) |  | -0.125 |  |
| ≥ 75% | 170（16.5%） | 53（20.2%） |  | 0.176 |  | 50 (17.4%) | 53(17.5%) |  | -0.069 |  |

Abbreviations: SD: standard deviation; Age-CCI: age-adjusted Charlson Comorbidity Index; BMI: body mass index; SOFA: Sequential Organ Failure Assessment; CRP: C-reaction protein; NIV: noninvasive mechanical ventilation；IMV: invasive mechanical ventilation；

PSM cohort: propensity score matched cohort.

Supplementary Table 3. Multivariable analysis of the ratio of an improvement of SOFA score decreased by more than 2 points for older subgroup (≥60 years old) after PSM

| Item | older Cohort | | | |
| --- | --- | --- | --- | --- |
|  | Patients with improvement ^a^ | Patients without improvement ^b^ | *P* value | Adjusted OR (95% CI) |
| Nmr/r plus standard therapy | 83 (60.1%) | 139 (47.3%) | 0.055 | 1.559 (0.990-2.455) |

a: Patients whose SOFA score decreased by more than 2 ( ≥ 2 ) points on Day 7.

b: Patients whose SOFA score decreased by less than 2 (< 2) points on Day 7.

In the new propensity score matched cohort, the heterogeneous covariates were eliminated. Using the adjusted regression to analysis the primary outcomes.

Supplementary Table 4. Multivariable analysis of the ratio of new intubation for older subgroup (≥60 years old) after PSM

| Item | older Cohort | | | |
| --- | --- | --- | --- | --- |
|  | Standard group | Nmr/r group | *P* value | Adjusted OR (95% CI) |
| The rate of new intubation  N (%) | 9 (34.6%) | 213 (52.5%) | 0.006 * | 0.152 (0.040-0.580)) |

* with significance and *P* ≤ 0.05.

In the new propensity score matched cohort, the heterogeneous covariates were eliminated. Using the adjusted regression to analysis the outcomes.
